# Carbon Source Influence on Extracellular pH Changes along Bacterial Cell-Growth

**DOI:** 10.3390/genes11111292

**Published:** 2020-10-30

**Authors:** Rubén Sánchez-Clemente, M. Isabel Guijo, Juan Nogales, Rafael Blasco

**Affiliations:** 1 Departamento de Bioquímica y Biología Molecular y Genética, Facultad de Veterinaria, Universidad de Extremadura, 10003 Cáceres, Spain; rubensc@unex.es (R.S.-C.); mguijo@unex.es (M.I.G.); 2 BioMic Research Group, Meat and Meat Products Research Institute (IProCar), Universidad de Extremadura, 10004 Cáceres, Spain; 3 Centro Nacional de Biotecnología, Department of Systems Biology, CSIC, 28049 Madrid, Spain; jnogales@cnb.csic.es; 4 Interdisciplinary Platform for Sustainable Plastics towards a Circular Economy-Spanish National Research Council (SusPlast-CSIC), 28040 Madrid, Spain

**Keywords:** pH homeostasis, systems biology, microbial ecology, biotechnology

## Abstract

The effect of initial pH on bacterial cell-growth and its change over time was studied under aerobic heterotrophic conditions by using three bacterial strains: *Escherichia coli* ATCC 25922, *Pseudomonas putida* KT2440, and *Pseudomonas pseudoalcaligenes* CECT 5344. In Luria-Bertani (LB) media, pH evolved by converging to a certain value that is specific for each bacterium. By contrast, in the buffered Minimal Medium (MM), pH was generally more stable along the growth curve. In MM with glucose as carbon source, a slight acidification of the medium was observed for all strains. In the case of *E. coli*, a sudden drop in pH was observed during exponential cell growth that was later recovered at initial pH 7 or 8, but was irreversible below pH 6, thus arresting further cell-growth. When using other carbon sources in MM at a fixed initial pH, pH changes depended mainly on the carbon source itself. While glucose, glycerol, or octanoate slightly decreased extracellular pH, more oxidized carbon sources, such as citrate, 2-furoate, 2-oxoglutarate, and fumarate, ended up with the alkalinization of the medium. These observations are in accordance with pH change predictions using genome-scale metabolic models for the three strains, thus revealing the metabolic reasons behind pH change. Therefore, we conclude that the composition of the medium, specifically the carbon source, determines pH change during bacterial growth to a great extent and unravel the main molecular mechanism behind this phenotype. These findings pave the way for predicting pH changes in a given bacterial culture and may anticipate the interspecies interactions and fitness of bacteria in their environment.

## 1. Introduction

Maintaining the intracellular concentration of protons within a certain range is very important in all biological systems because the structure/function of proteins and other macromolecules depends on pH. Moreover, pH affects the kinetic and thermodynamic parameters of all biochemical reactions in which protons are involved as reactants. Especially important is respiration, where the proton motive force (PMF) couples the electron transport chain to ATP synthesis [1] (oxidative phosphorylation). In eukaryotes, mitochondria and chloroplasts are surrounded by the cytoplasm, which exhibits strict pH homeostasis [2]. The scenario is quite different in bacteria, where PMF is produced in the inner membrane that separates the cytosol and the periplasm in Gram-negative bacteria. In this sense, bacteria are more exposed to extracellular pH changes than eukaryotes.

Bacteria thrive in different habitats and display different levels of tolerance to environmental pH. In fact, this is the basis for classifying bacteria into acidophiles (pH 1–3), alkalophiles (pH 10–13), and neutrophiles (pH 5.5–9). However, as in Eukarya, the intracellular pH of bacteria is close to neutrality and remains almost constant in order to preserve metabolic capacity and cellular integrity. Bacterial pH homeostasis includes diverse mechanisms for direct sensing and adapting to extracellular pH [3]. In addition to ATP synthesis, bacterial PMF can be utilized to actively transport metabolites through the inner membrane (i.e., MSF [4]), as well as macromolecules through the outer membrane using the TonB-dependent transport systems [5]. PMF also provides energy for driving the bacterial flagellar motor [6].

The extracellular pH may change due to several factors. Growing *E. coli* cells in LB-medium were shown to cause progressive changes in pH and sugar availability, which in turn influence both the cellular heterogeneity within the microbial community and the microbial population’s gene-expression profile [7]. The variation of pH has been shown to regulate genes involved in catabolism and metabolite transport, including multidrug transporters [8]. Therefore, pH changes affect bacterial susceptibility to antibiotics for two reasons, the presence of a transporter in the membrane, and, in some cases, the permeability of the antibiotic as a function of pH [9,10]. The chemical composition of the culture medium defines its initial pH, but metabolic reactions during growth phase and adaptation to the medium trigger changes in extracellular pH.

One of the main components of the culture medium is the carbon source. Heterotrophic bacteria can use a great variety of organic compounds as carbon source, either natural or xenobiotic [11]. Some bacterial groups, including strains from the Pseudomonaceae genus, are able to use a plethora of carbon sources ranging from highly reduced, such as alkanes or alcohols, to more oxidized carbon sources, such as di- and tri-carboxylic acids of the Krebs cycle, or even the more oxidized formic acid [12]. Through a series of peripheral reactions, these compounds are converted into others included in the core network, which includes a small part of the metabolic network that is strongly connected to the central routes [13]. Therefore, growth of a given bacterial strain may disturb the growth of neighboring strains sharing the same ecological niche and these changes may decide the fate of the entire bacterial population [14]. From this point of view, understanding pH homeostasis may have implications and applications in such diverse fields as bioremediation [15], metabolite production [16], and the behavior of pathogenic bacteria in terms of their direct interaction with the host and the development of methods to control food-borne pathogens [17,18]. Moreover, although strictly speaking, ageing is an intrinsic process, external perturbations such as drastic changes in external pH associated to a given carbon source could definitely guide more evident ageing phenotypes [19].

The change of extracellular pH can be predicted “in silico” using genome-scale metabolic models (GEMs) because, ultimately, the extent of the metabolic network is limited by the reactions encoded in its genome and the composition of the medium. GEMs contain detailed information on the target organism and the exact reaction stoichiometry (including protons), the relationships between genes, proteins, and reactions (GPR), as well as the biochemical and physiological data available subject to specific nutritional scenarios. The aim of this work was to study the influence of the carbon source on pH-change during aerobic growth of some model heterotrophic bacteria. Consequently, the results obtained using GEM models of *Pseudomonas putida* KT2440 [20], *Escherichia coli* K-12 MG1655 [21], and *Pseudomonas pseudoalcaligenes* CECT 5344 (submitted for publication) were confronted with experimental results. The predicted results strongly agree with pH variations observed in vivo, giving insights on the molecular mechanisms behind pH change. These data could be useful for optimizing media composition for biotechnological applications such as the production of chemicals and predicting biological fitness of bacteria in their ecological niches.

## 2. Materials and Methods

Liquid Culture media: Luria–Bertani media (LB) [22] was adjusted to pH 6, 7, 7.5, 8, 8.25, and 9 with NaOH before autoclaving. M63 minimal media [23] supplemented with glucose, citrate, propionate, octanoate, fumarate, 2-oxoglutarate, 2-furoate, or glycerol as carbon source, at a final concentration of 4.0 g/L, were adjusted to the desired pH with KOH before autoclaving. The cells were cultured aerobically in a rotary shaker at 200 rpm and 30 °C for *Pseudomonas*, and 37 °C for *Escherichia coli*. Bacterial cell growth was followed turbidimetrically by measuring the absorbance of 1 mL culture samples in 1 cm path plastic cuvettes at 600 nm, using a Helios Epsilon colorimeter (Thermo Scientific, Waltham, MA, USA). The growth curves were run in triplicate, giving similar results. Therefore, in order to clarify the graphs, they show the results from a single representative experiment. The pH of the culture media was measured with a conventional pH electrode (CRISON, Madrid, Spain).

Genome-scale metabolic reconstruction for *P. pseudoalcaligenes* was performed using a defined protocol [24], which resulted in model iRS1006 (unpublished). The genome-scale models we used for *Escherichia coli* and *Pseudomonas putida* were previously published as *i*JO1366 [21] and iJN1411 [20,25], respectively. The “in silico” assessment of proton exchange as a function of the carbon source using GEMs was done as previously described [26]. To study the reactions involved in the pH changes using different carbon sources for a defined bacterial strain, the fluxes were normalized against the biomass reaction using glucose as carbon source. The flows were ordered following the absolute difference of the expected normalized flow against the predicted flow (Supplementary Table S1). The exchange fluxes for the carbon source, CO_2_, pH (H^+^), H_2_O, and O_2_ were obtained directly from the table.

## 3. Results

Among the many factors affecting changes in extracellular pH with bacterial growth, this work focused on the influence of the carbon source. Therefore, a set of experiments was designed to separately determine how initial pH and the bacterial strains themselves influence changes in pH. The influence of bacterial strain was tested using either complex media or minimal medium with glucose as carbon source for each of the bacterial strains. The influence of pH was assessed using the same medium and modifying the initial pH. On the other hand, the influence of the media was assessed by changing the carbon source in defined minimal media for a single strain.

### 3.1. Influence of the Initial pH

To test the influence of initial pH and bacterial strain it was necessary to choose culture media in which all bacterial strains could thrive. The Luria–Bertani (LB) medium was the complex medium of choice, whereas the M63 medium, with glucose as carbon source, was used to test pH changes in minimal medium. Since both *E. coli* and *P. putida* are neutrophilic organisms, the initial pH of the media was set to 6, 7, or 8. By contrast, since *P. pseudoalcaligenes* is able to grow under alkaline conditions [27], the initial pH for this strain was set to 7.5, 8.25, and 9.

#### 3.1.1. Change of Extracellular pH in Complex (LB) Media

First, we monitored cell growth and pH changes along the growth curve at different initial pH in Luria–Bertani medium. Since this is not a defined medium, it was not possible to simulate bacterial metabolism and the change of pH by using genome-scale metabolic models (GEM). Therefore, it was not possible to confront the experimental results with those obtained theoretically.

**Figure 1:**
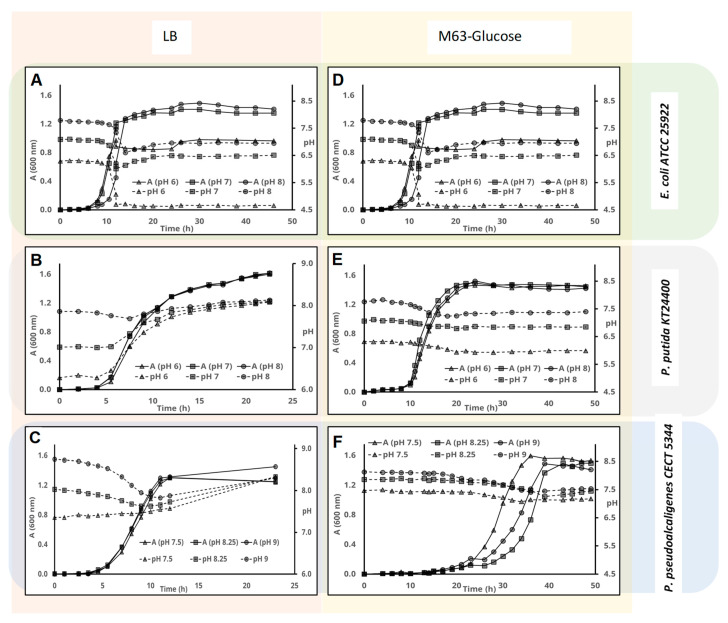
Influence of the initial pH on pH change in different bacterial strains. Cells were inoculated in either Luria-Bertani (LB) complex medium (panels (**A**–**C**)) or defined minimal M63 medium with glucose as C-source (panels (**D**–**F**)). The pH of the medium (discontinuous lines) and cell-growth (A_600_ nm, continuous lines) were measured at the indicated times for *E. coli* ATCC 25,922 (panels (**A**,**D**)), *Pseudomonas putida* KT2440 (panels (**B**,**E**)), and *Pseudomonas pseudoalcaligenes* CECT 5344 (panels (**C**,**F**)).

In the case of *E. coli* (Figure 1A), pH did not change during the lag phase in LB medium, but it suddenly dropped during the exponential growth phase. The higher the initial pH, the more dramatic was the pH drop. However due to further alkalinization of the media, pH converged to 8 at the end of the exponential phase, and finally to around 8.5, irrespective of the initial pH. Interestingly, these different pH-change paths did not impact on final cell density, which was almost the same in all cases.

When cell-growth (A_600nm_) and pH change was monitored for *P. putida* in LB media at different initial pH, we found that, as with *E. coli*, pH did not change during the latency phase. However, the pH of the culture media increased during the exponential phase, with alkalinization inversely proportional to initial pH and converging to around 7.5 (Figure 1B). Alkalinization continued during the stationary phase until a peak was reached at around 8. As for *E. coli*, the final A_600nm_ were very similar in the three-culture media.

When the behavior of *P. pseudoalcaligenes* was analyzed, we observed that while pH did not change during the latency phase, it decreased slowly during the exponential growth phase when the initial pH was 8.25 or 9, and it increased slowly when the initial pH was 7.5 (Figure 1C). pH converged to a narrow range towards the end of the exponential phase. Alkalinization of the medium continued during the stationary phase, reaching a peak around 8.5–9. The final A_600nm_ were similar irrespective of the initial pH, although it was slightly higher at pH 9.

#### 3.1.2. pH Change and Cell-Growth in MM with Glucose as Carbon Source

Glucose can be used by many microorganisms as carbon source. When *E. coli* was cultured in glucose, the pH did not change during the latency phase, which was a bit longer at pH 8. At the beginning of the exponential phase, the pH suddenly dropped. At pH 7 and 8 the pH drop was around one unit, but further cell growth restored the pH up to pH 6.5 and 7, respectively. Nevertheless, the pH drops in the culture media with initial pH 6 was close to two pH units, thus inhibiting further bacterial growth and re-alkalinization of the medium (Figure 1D).

In the case of *P. putida* and *P. pseudoalcaligenes*, the pH-change paths were much more stable than in *E. coli*. In addition, we found that the duration of the latency phase was much longer in *P. pseudoalcaligenes* (Figure 1C,E). In any case, the pH did not change during this phase, but the pH of the culture media decreased slightly for both strains along the exponential phase with glucose as carbon source. During the stationary phase, the pH did not change and the A_600nm_ were very similar for all cultures.

### 3.2. Influence of the Carbon Source on pH Change

The type of carbon sources utilized by a certain bacterial strain is fundamentally limited by its genotype, which determines the metabolic routes available for their assimilation. Glucose is a reliable carbon source for many bacteria, including all strains utilized in this work, whereas other carbon sources are species-specific. Table 1 contains a summary of the carbon sources used in this study, classified according to their oxidation number (ON). The ON of the carbon source is its theoretical valence, which is obtained by assigning a value of −2 to oxygen and +1 to hydrogen. The standard enthalpy of combustion per carbon atom decreases as the oxidation number increases. The table also contains the adjusted oxidation reactions that can be used to estimate the expected O_2_ consumption, and H_2_O and CO_2_ production, per carbon atom.

#### 3.2.1. Growth Curve and pH Change of *E. coli* ATCC 25,922 with Different Carbon Sources

**Figure 2:**
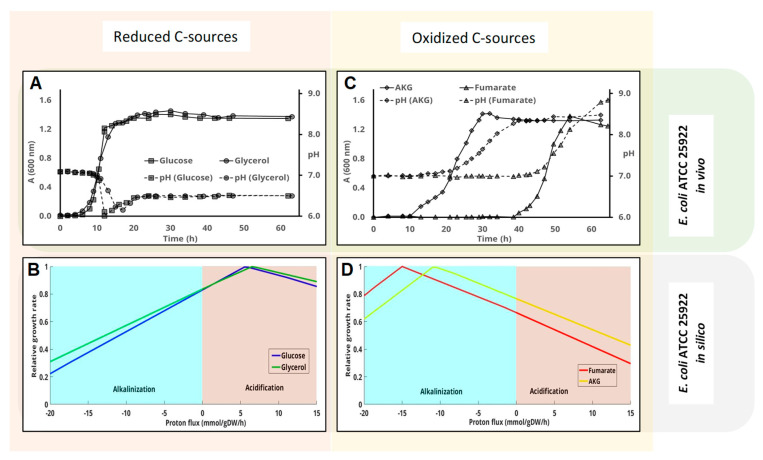
Influence of the carbon source on the pH change in *E. coli* ATCC 25,922 cultures. Cells were inoculated in M63 media containing different carbon sources. Cell-growth (solid lines-A(600 nm)) and pH change (dashed lines) were measured at the indicated times. Glucose and glycerol, which are considered reduced carbon sources, are plotted together (panel (**A**)), whereas panel (**C**) shows the results obtained using the more oxidized carbon source fumarate and 2-oxoglutarate. Panels (**B**,**D**) represent the predicted influence of proton flux (negative implies uptake and therefore alkalinilization of the medium) on the growth rate using the genome scale metabolic model of this strain as indicated in the Materials and methods section.

According to previous sections, pH changes could be a consequence of the oxidation state of the carbon source. Therefore, we tested this hypothesis by using the available GEMs for the three bacteria above. GEMs can be used to estimate pH change during bacterial growth by monitoring the flux through proton exchange reaction while maximizing growth. A negative flux through this reaction is indicative of active proton uptake, thus resulting in external media alkalinization and vice versa [26]. The carbon sources for each assay were selected on the basis of their ON. Considering that in nature, carbon’s ON range from −4 to +4, carbon sources with an oxidation number equal to or lower than zero can be arbitrarily considered reduced. In the case of *E. coli*, the selected carbon sources were, in addition to glucose (oxidation number 0), glycerol, which is more reduced than glucose (ON −0.66), and two oxidized carbon sources: 2-oxoglutarate and fumarate (ON 0.8 and 1, respectively) (Table 1). At maximum growth rates, the *E. coli* (*i*JO1366) model predicted a significant acidification of the external medium when using reduced carbon sources (Figure 2B). By contrast, maximum growth rate using oxidized carbon sources requires the alkalinization of the medium (Figure 2C). As a matter of fact, the higher proton uptake flux (alkalinization) was predicted using fumarate (the most oxidized carbon source tested) as the sole carbon source. The reader should note that the “in silico” predictions (Figure 2B,D) compute growth rate (Y axis) as a function of fixed proton exchange values (*X*-axis), where positive and negative values indicate proton secretion and uptake, respectively. On de contrary, Panels A and B are time-course experimental results.

The results of “in vivo” laboratory experiments and GEM predictions were in good agreement. Initial pH was always 7 for this set of experiments. As expected, bacterial growth with reduced carbon sources (glucose and glycerol) resulted in the final acidification of the medium (Figure 2A). As previously shown, growth with glucose proceeded with a reversible drop during the exponential growth phase that simulates peak growth rate in the exponential phase. This was not predicted using GEM. By contrast, growth at the expense of fumarate and 2-oxoglutarate resulted in a strong alkalinization of the medium (Figure 2C). The highest alkalinization (up to pH 9) was recorded with the most oxidized carbon source—fumarate (Figure 2C), as predicted by the model (Figure 2D), the lag phase was also longest with fumarate (almost 35 h, Figure 2C). Final optical density was similar for all carbon sources. However, it is remarkable that the final optical density was a bit higher with reduced carbon sources, and was highest with glycerol, followed by glucose, fumarate, and 2-oxoglutarate.

#### 3.2.2. Growth Curve and pH Change of *P. putida* KT2440 with Different Carbon Sources

**Figure 3:**
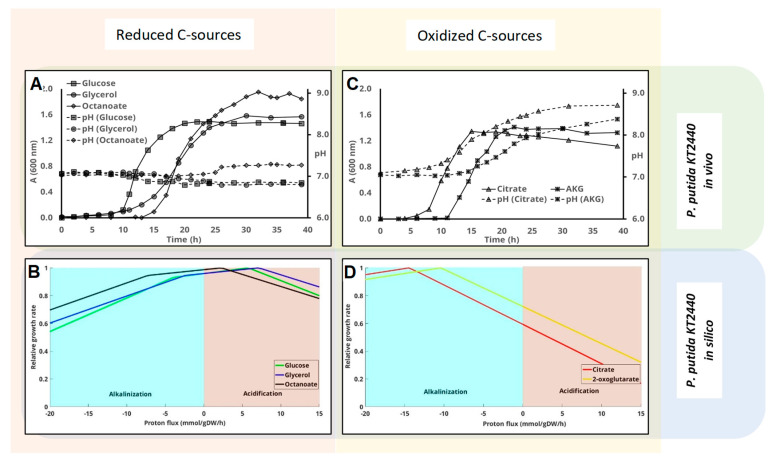
Influence of the carbon source on the pH change in *P. putida* KT2440 cultures. Cells were inoculated in M63 media containing different carbon sources. Cell-growth (solid lines, A_600 nm_) and pH values (dashed lines) were measured at the indicated times. Glucose, glycerol, and octanoate, which are considered reduced carbon sources, are plotted together in panel (**A**), whereas panel (**C**) shows the results obtained using more oxidized carbon sources (citrate and 2-oxoglutarate). Panels (**B**,**D**) represent the predicted influence of proton flux (negative implies uptake and therefore alkalinization of the medium) on the growth rate using the genome scale metabolic model of this strain as indicated in the Materials and Methods and Material section.

The metabolic model of *P. putida* KT2440 (*i*JN1411) was used to predict the optimum pH for growth with octanoate (ON = −1.5), glycerol (ON = −0.66), glucose (ON = 0), 2-oxoglutarate (ON = +0.8), and citrate (ON = +1). Glucose, glycerol, and 2-oxoglutarate were used as carbon sources to compare the results with those previously obtained for *E. coli*. Nevertheless, citrate was used instead of fumarate because, although both have the same oxidation state, citrate is a preferable carbon source for *P. putida*. Finally, octanoate was also used because it is even more reduced than glycerol and both can be used by *P. putida* and *P. pseudoalcaligenes* CECT 5344 for bioplastic production [29,30,31]. The model predicted the acidification of the medium with the reduced carbon sources octanoate, glycerol, and glucose (Figure 3B) and alkalinization of the cultures with 2-oxoglutate and citrate (Figure 3C). Moreover, the model predicted that the most alkalinizing carbon source is citrate (Figure 3D).

GEM predictions were validated in the laboratory. Since this bacterium is neutrophilic, the initial pH was always 7, as described above for *E. coli*. Culture media supplemented with glucose and glycerol became a little bit acidified during the exponential phase, whereas pH remained virtually constant in culture media with octanoate (Figure 3A). By contrast, culture media containing 2-oxoglutarate or citrate as carbon sources underwent a clear alkalinization that began during the exponential phase and continued until the end of the stationary phase (Figure 3C). As observed for *E. coli* (Figure 2), alkalinization was greater with citrate than with 2-oxoglutarate, which is fully compliant with the “in silico” results (Figure 3D). The highest absorbance was reached with octanoate, followed by glycerol, glucose, 2-oxoglutarate, and citrate.

#### 3.2.3. Growth Curve and pH Change of *P. pseudoalcaligenes* CECT 5344 with Different Carbon Sources

**Figure 4:**
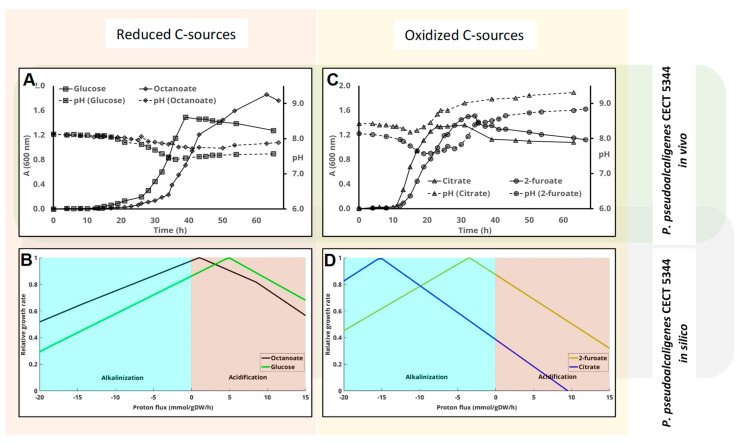
Influence of the carbon source on pH change in *P. peudoalcaligenes* CECT 5344 cultures. Cells were inoculated in M63 media containing different carbon sources. Cell-growth (solid lines, A_600 nm_) and pH change (dashed lines) were measured at the indicated times. Glucose and octanoate, which are considered reduced carbon sources, are plotted together (panel (**A**)), whereas panel (**C**) shows the results obtained using more oxidized carbon sources (citrate and 2-furoate). Panels (**B**,**D**) represent the predicted influence of proton flux (negative implies uptake and therefore alkalinization of the medium) on the growth rate using the genome scale metabolic model of this strain as indicated in the M&M section.

Simulation of the growth rate vs. proton exchange by means of *i*RS1006 predicts the acidification of the medium with glucose and octanoate (Figure 4B) and its alkalinization with furoate and citrate (Figure 4D). The same carbon sources as for *P. pudida* KT24440 were used, replacing 2-oxoglutarate with furoate just to expand the validity of this study using different carbon sources that have been found to be used by this strain [32]. Both furoic acid and 2-oxoglutarate are more oxidized than glucose (Table 1). Nevertheless, whilst 2-oxoglutarate is a metabolite directly involved in the Krebs cycle, the metabolism of furoic acid in *P. pseudoalcaligenes* is much more complex [32].

Considering the physiology of the *P. pseudoalcaligenes* strain, the starting pH for the “in vivo” experiments was set to 8 instead of 7. The metabolism of both citrate and furoate by *P. pseudoalcaligenes* resulted in the alkalinization of the medium (Figure 4C). This alkalinization was higher with citrate than with furoate, reaching a final pH of 9.5. Meanwhile, the metabolism of the more reduced metabolites glucose and octanoate ended up with the acidification of the cultures (Figure 4A). The same occurred with glycerol, but after a longer lag period (not shown). The carbon source that returned peak absorbance was octanoate, followed by glucose, furoate, and citrate.

Overall, the experiment described above strongly suggests that the ON of the carbon source is a key factor in determining the final pH of the medium after bacterial growth. As indicated, pH changes in the medium can be predicted using GEMs. Therefore, the next goal in this work was to examine the main metabolic fluxes in order to understand the metabolic basis of this fact. Only three carbon sources were utilized for this purpose, and *P. putida* as model organism.

### 3.3. Analysis of pH Change and Detailed Metabolic Flux Balance Analysis of P. putida KT2440 with Different Carbon Sources

The catabolism of a certain carbon source depends on the genotype of the bacterium, but it usually converges to central metabolic routes. Under aerobic conditions, the main central oxidative pathway is the Krebs cycle, rendering CO_2_ and reducing power. The reducing power can be used both as “fuel” for ATP synthesis through oxidative phosphorylation or as a source of electrons for biosynthetic reactions (mainly as NAD(P)H). The aerobic oxidation of the carbon sources always renders CO_2_ (ON +4). Therefore, an organic compound’s reducing power can be theoretically calculated by adjusting its hydration reaction to render CO_2_ and reducing power (H_2_), as reflected in the following table (Table 2).

Obviously, H_2_ is not metabolically produced by aerobic heterotrophic bacteria, but it is chemically equivalent to NAD(P)H. If coupled to the oxidative phosphorylation, the reducing power drives ATP synthesis powered by the highly exergonic reaction ½ O_2_ + NADH + H^+^ → H_2_O. This reaction coupled to the hydrolytic dehydrogenation of the carbon source (Table 2) are processes equivalent to the combustion of each compound (Table 1). For example, in the case of glucose:

C_6_H_12_O_6_ + 6 H_2_O → 6 CO_2_ + 12 H_2_

12 H_2_ + 6 O_2_ → 12 H_2_O

C_6_H_12_O_6_ + 6 O_2_ → 6 CO_2_ + 6 H_2_O

**Figure 5:**
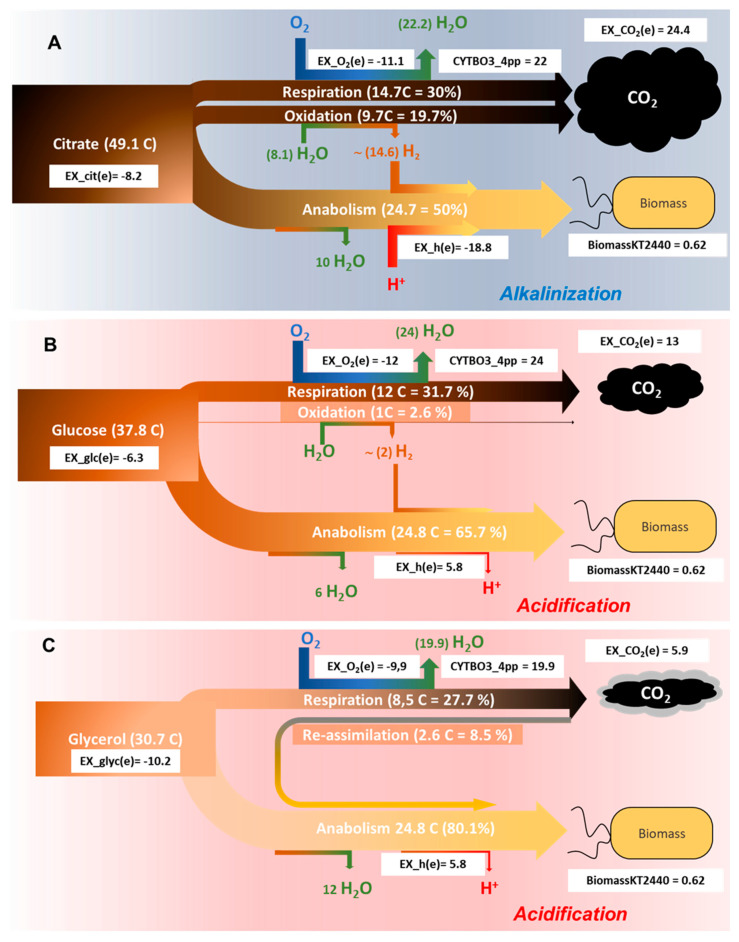
Carbon-flow distribution in *P. putida* 2440 (iJN1411) as a function of the carbon source. Cell growth (biomassKT2440) was normalized as indicated in M&M. The white rectangles represent the normalized flow through the indicated reactions. The total amount of citrate (**A**), glucose (**B**), or glycerol (**C**) is divided into two main streams, one rendering biomass (anabolism, lower part of each scheme) or CO_2_ (catabolism, upper part). The percentage and number of carbons through respiration was calculated from the consumed O_2_ (ex_o2(e)) according to Table 1. The difference between the produced CO_2_ (ex_co2(e)) and that expected from respiration (Table 1) is re-assimilated in the case of glycerol (**C**) or oxidized according to Table 2, rendering reducing power. CYBO3_4pp: Cytochrome *c* oxidases, *bo3*-type).

Given that the majority of the oxygen consumed under aerobic conditions is due to respiration (see below), the amount of CO_2_ produced per consumed O_2_ can be anticipated considering the stoichiometry of the reactions represented in Table 1. In order to understand the flux distribution of carbon as a function of the carbon source, the predicted metabolic fluxes for *P. putida* were calculated using *i*JN1411 [25] (Supplementary Table S1). The carbon sources employed were glucose (ON 0), citrate (ON +1), and glycerol (ON −0.66). The results obtained are schematized in Figure 5. Fluxes were normalized according to the biomass reaction (BiomassKT2440) with glucose as carbon source and sorted according to the difference between the normalized and the predicted flow for each carbon source (Supplementary Table S1). Since glucose was selected as the reference carbon source, it is represented in the central panel (Figure 5B). The model was adjusted for an intake of glucose of 6.3 mmol g^−1^ h^−1^ (37.8 glucose carbons per dry gram of biomass and hour) (Figure 5B). The predicted uptake of oxygen (EX_o2(e)) was 11.99 mmol g^−1^ h^−1^, while the flux through terminal oxidase (cytochrome *bo* terminal oxidase-CYTBO3_4pp) was almost double (23.96). Considering that the flux through the terminal oxidase is adjusted per electron pair, and that one molecule of oxygen is equivalent to two electron pairs, this result reflects, as expected, that under these conditions respiration is the main oxygen sink. Therefore, and taking into account the stoichiometry shown in Table 1, the expected CO_2_ produced is 12 carbons (one CO_2_ per O_2_ in the case of glucose). Nevertheless, the normalized exchange of CO_2_ was 13 mmol g^−1^ h^−1^ (Supplementary Table S1). Thus, we must assume that the reducing power of an extra glucose carbon was used in anabolic reactions and/or cell maintenance. Provided that ON is zero for glucose, this is equivalent to two electron pairs (Table 2, Figure 5B). In total, 34.4% of the consumed glucose is converted into CO_2_, and the rest (65.6%) into biomass (Figure 5B).

The normalized exchange of citrate was 8.2 (EX_cit(e) = −8.2). This means that the number of carbons needed to obtain the same biomass from citrate or glucose was 49.14, i.e., 30% more (Figure 5A). By contrast, the same biomass can be obtained with 30.7 carbons of glycerol (EX_glyc(e)) = −10.2), i.e., 81% less than that what is required with glucose (Figure 5C). In the case of citrate, oxygen consumption during respiration was 11.1 mmol g^−1^ h^−1^, equivalent to 14.8 mmol g^−1^ h^−1^ of CO_2_ according to Table 1. Since the exchange of CO_2_ predicted in the model was much higher (EX_co2(e) = 24.4), this means that 9.7 mmol g^−1^ h^−1^ of CO_2_ are metabolically generated by reactions equivalent to its hydration (Table 2). In the case of citrate, 9.7 CO_2_ will generate 14.7 electron pairs (H_2_) (Table 2) that are utilized in the transformation of citrate into biomass, as schematized in Figure 5A. By contrast, the production of CO_2_ with glycerol as carbon source was less than expected given the level of oxygen consumption (Figure 5C, Table 1). Specifically, consumption of 9.9 mmol g^−1^ h^−1^ of O_2_ should generate 8.6 mmol g^−1^ h^−1^ of CO_2_ during the oxidation of glycerol (Table 1). Since only 5.9 are produced (EX_co2(e)= 5.9), we must assume that 2.7 mmol g^−1^ h^−1^ of CO_2_ became re-assimilated as biomass (Figure 5C).

It is also interesting to note that, under normalized flow through the ATP synthase is almost equal for glucose and glycerol (32 mmol g^−1^ h^−1^), whereas it is approximately 35 mmol g^−1^ h^−1^ for citrate (Supplementary Table S1). This result indicates that synthesis of biomass from citrate requires extra ATP. Since respiration is similar for glucose and citrate, the extra flow through the ATP synthase will take place with protons derived from the medium, thus contributing to its alkalization. Figure 5 shows total exchange of protons during this process and the one discussed below. The amount of carbon needed to obtain the same biomass increases with ON, which is reflected as an increase of CO_2_ production and intake of protons, whereas the flow through respiration does not change significantly (Figure 5). Remarkably, predicted CO_2_ produced from the most reduced carbon source is less than that expected by oxygen uptake.

The exchange reactions represented in Figure 5, including protons, are those estimated by FBA to maximize the biomass reaction at a fixed intake of the corresponding carbon source, under stationary conditions. Therefore, a negative exchange of protons (Figure 5A) is represented by a converging arrow associated to biomass production, thus causing an acidification of the medium. By contrast, a positive value of the exchange reaction of protons means production of protons associated to biomass generation, which in turn acidifies the medium. In this scheme, the proton exchange reaction is associated to biomass production, not to specific reactions as will be discussed below.

## 4. Discussion

There are many studies describing how the pH of the medium affects bacterial growth, the production of certain metabolites and their ecological fitness [3,14]. Motile bacteria migrate to areas where pH is optimal by means of a mechanism recently described in *Bacillus* [33,34]. In fact, bacterial pH homeostasis has been monographically treated in some instances [35], but the change of the pH of the medium during microbial growth has been studied to a lesser extent [36]. Early papers demonstrated that a change in external pH was followed by an alteration in the enzymatic constitution of the cells attempting to counter the external change [37]. Later on, it has been shown that pH changes affect the expression of specific genes [7], and that some bactericidal antibiotics kill via an increase in cytoplasmic pH [38]. Therefore, controlling pH homeostasis may protect against stresses, including some bactericidal antibiotics [39]. These findings open a new mechanism to fight against some pathogens showing continuous rise in antibiotic resistance [40]. In this manuscript the predicted change in pH was studied taking advantage of well-curated bacterial GEMs and the results were confronted with empirical data of pH-change in different culture media. Metabolic models stoichiometrically adjust the metabolic flux required for balanced cell growth. Since the application of GEMs requires defined culture media with well-known composition, its extrapolation to complex media, such as LB, is challenging [41]. Nevertheless, the utilization of LB allows checking the influence of the phenotype of many bacteria. In general, pH changes in LB media were higher than in M63 media (Figure 1), probably because the LB medium is not buffered. LB is essentially a carbohydrate-free mixture of amino acids and small peptides routinely used in many microbiology laboratories. In *E. coli*, the initial pH did not impact on final cell density in LB media (Figure 1A), despite having shown that small changes in pH and sugar availability have an effect on both cellular heterogeneity and the gene-expression profile [7]. In defined media it was shown that the level of pH change depends on the amount of some nutrients (glucose and urea) [14]. The transient acidification of the medium by *E. coli*, either in complex or defined medium, has been extensively studied and can be explained in the context of the acetate switch [42], a process that occurs even under unlimited-oxygen conditions [43]. This behavior was also experimentally observed in this work, demonstrating that when the initial pH was 6 in glucose-minimal medium, the acidification of the media irreversibly prevents further cell-growth and, consequently, the re-alkalinization of the cultures. Nevertheless, the model cannot predict transient accumulation of metabolites because the nature of Flux Balance Analysis in which steady state assumption is assumed. Therefore, pH changes due single charged molecules other than protons is out of the scope of our ′′in silico′′ analysis.

The genome-scale metabolic reconstruction collects all known metabolic capabilities of an organism as defined by its genome. These models can be transformed into a mathematical format, thus enabling phenotype computing under specific environmental conditions [44]. Environmental and internal parameters can be changed over a finite range of numerical values to study their influence on the system. *P. putida* has been utilized as a model and functional chassis for industrial biocatalysis [45], and a well-curated model of its metabolism is available [25]. For these reasons, it was used here to acquire a deeper understanding of the molecular mechanisms behind the pH changes, which is summarized in Figure 5. Nevertheless, the same conclusions were obtained by using the other two bacterial strains (not shown).

Carbon sources serve as carbon and energy source for heterotrophic bacteria. Upon entering the cell, part of the carbon source is transformed into biomass (anabolism) at the expense of the energy obtained from another fraction that is transformed into CO_2_ (catabolism). The more reduced a compound is, the more energy can be obtained from its oxidation (Table 1), and more proliferation can be achieved (Figure 2, Figure 3 and Figure 4). The stoichiometry and the composition of living organisms [46] give rise to a molecular formula for the biomass in which the carbon will have a specific oxidation number. The composition of biomass has been studied in many microorganisms, and the oxidation number of carbon in bacteria is close to zero in the negative range (−0.27 in average) [47,48]. The same conclusion can be obtained from Figure 5B because the produced CO_2_ is almost the same as what might be expected form oxygen exchange. Therefore, in order to simplify the stoichiometric numbers, we can consider the transformation of a certain carbon source into glucose as its transformation into biomass. For example, the adjusted transformation of citrate into glucose can be adjusted as:

$$4C_{6}H_{5}O_{7}^{3-}+3H^{+}+2H_{2}O\to 3C_{6}H_{12}O_{6}+6{\mathrm{CO}}_{2}$$

The stoichiometry of this reaction reveals that the biochemical transformation of a certain oxidized carbon source such as di- or tri-carboxylic acids, into biomass can only occur with its partial oxidation to CO_2_ to obtain reducing power for the conversion of citrate into biomass. Moreover, alkalinization of the medium due to proton consumption became evident. The production of CO_2_ is associated to the generation of reducing power because the analysis of the predicted flows shows that the reducing power does not take the respiration “wire”, as shown in Figure 5. The generation of biomass needs both reducing power and chemical energy in the form of ATP. As a matter of fact, the predicted flow through ATP synthase is more than double with citrate as carbon source if compared to glucose (Supplementary Table S1). Since this reaction consumes four protons, presumably it is partially responsible for the alkalinization of the medium. Other reactions contributing to the consumption of protons are the formation of biomass itself, the symport of the negatively charged carbon source with protons (or sodium), the increase of flow through proton-consuming reactions, such as the oxaloacetate decarboxylase, and the decrease of flow through proton-releasing reactions, such as the phosphoenolpyruvate carboxylase (Supplementary Table S1). In photosynthetic organisms, which utilize HCO_3_^−^ as carbon source, alkalinization of the medium has also been described [26].

On the contrary, the generation of biomass using reduced carbon sources, such as glycerol, consumes CO_2_ according to the following adjusted reaction:

$$12C_{3}H_{8}O_{3}+6{\mathrm{CO}}_{2}\to 7C_{6}H_{12}O_{6}+6H_{2}O$$

The required reducing power for assimilating CO_2_ is obtained from the reduced carbon source itself, but not affecting the extracellular pH, as experimentally demonstrated in this work. Since glycerol is a by-product of the production of biodiesel, it is a value-added resource for biomass and bioplastics production. The production of biomass from glycerol seems to contribute to lower the levels of CO_2_ in the atmosphere if compared to its combustion (Table 1).

In conclusion, the results obtained in this work indicate that pH change along bacterial cell-growth strongly depends on the composition of the medium; oxidized carbon sources will favor the alkalinization of the medium, whilst reduced substrates will drive a slight acidification. These conclusions may have great impact in clinical, industrial, and environmental biotechnology, as well as food technology.

## Figures and Tables

**Table 1:** Properties of the chemical compounds used as carbon sources in the present manuscript.

Carbon Source	Formula	ON ^a^	Adjusted Oxidation Reaction	Standard Enthalpy of Combustion ^b^	
				ΔH^0^ (kJ·mol^−1^) ^c^	ΔH^0^ (kJ·Carbon^−1^) ^d^
Octanoic acid	C_8_H_16_O_2_	−1.5	C_8_H_16_O_2_ + 11 O_2_ → 8 CO_2_ + 8 H_2_O	−798	−600
Glycerol	C_3_H_8_O_3_	−0.66	C_3_H_8_O_3_ + 3.5 O_2_ → 3 CO_2_ + 4 H_2_O	−1654	−551
Glucose	C_6_H_12_O_6_	0	C_6_H_12_O_6_ + 6 O_2_ → 6 CO_2_ + 6 H_2_O	−3165	−528
2-Furoic acid	C_5_H_4_O_3_	0.4	C_5_H_4_O_3_ + 4.5 O_2_ → 5 CO_2_ + 2 H_2_O	−2041	−408
2-oxoglutaric acid	C_5_H_6_O_5_	0.8	C_5_H_6_O_5_ + 4 O_2_ → 5 CO_2_ + 3 H_2_O	−1822	−364
Fumaric acid	C_4_H_4_O_4_	1	C_4_H_4_O_4_ + 3 O_2_ → 4 CO_2_ + 2 H_2_O	−1334	−333
Citric acid	C_6_H_8_O_7_	1	C_6_H_8_O_7_ + 4.5 O_2_ → 6 CO_2_ + 4 H_2_O	−1960	−327

^a^ Oxidation number (ON).^. b^ Data from Haynes et al. (2017) [28]. ^c^ Combustion enthalpy per mol. ^d^ Combustion enthalpy per carbon atom.

**Table 2:** Reducing power, expressed as H_2_, of the different carbon sources used in this work.

Carbon Source	Formula	ON ^a^	Adjusted Hypothetical Hydration Reaction	Normalized Reducing Power(H_2_/Carbon)
Octanoic acid	C_8_H_16_O_2_	−1.5	C_8_H_16_O_2_ + 14 H_2_O → 8 CO_2_ + 22 H_2_	2.75
Glycerol	C_3_H_8_O_3_	−0.66	C_3_H_8_O_3_ + 3 H_2_O → 3 CO_2_ + 7 H_2_	2.33
Glucose	C_6_H_12_O_6_	0	C_6_H_12_O_6_ + 6 H_2_O → 6 CO_2_ + 12 H_2_	2
2-Furoic acid	C_5_H_4_O_3_	0.4	C_5_H_4_O_3_ + 7 H_2_O → 5 CO_2_ + 9 H_2_	1.8
2-oxoglutaric acid	C_5_H_6_O_5_	0.8	C_5_H_6_O_5_ + 5 H_2_O → 5 CO_2_ + 8 H_2_	1.6
Fumaric acid	C_4_H_4_O_4_	1	C_4_H_4_O_4_ + 4 H_2_O → 4 CO_2_ + 6 H_2_	1.5
Citric acid	C_6_H_8_O_7_	1	C_6_H_8_O_7_ + 5 H_2_O → 6 CO_2_+ 9 H_2_	1.5

^a^ Oxidation number.

## Supplementary Materials

The following are available online at https://www.mdpi.com/2073-4425/11/11/1292/s1, Table S1: Normalized flows.

## References

[1] Kühlbrandt W., Davies K.M. (2016). Rotary ATPases: A New Twist to an Ancient Machine. Trends Biochem. Sci..

[2] Casey J.R., Grinstein S., Orlowski J. (2009). Sensors and regulators of intracellular pH. Nat. Rev. Mol. Cell Biol..

[3] Krulwich T.A., Sachs G., Padan E. (2011). Molecular aspects of bacterial pH sensing and homeostasis. Nat. Rev. Microbiol..

[4] Pao S.S., Paulsen I.T., Saier M.H. (1998). Major facilitator superfamily. Microbiol. Mol. Biol. Rev..

[5] Noinaj N., Guillier M., Barnard T.J., Buchanan S.K. (2010). TonB-dependent transporters: Regulation, structure, and function. Annu. Rev. Microbiol..

[6] Nirody J.A., Sun Y.-R., Lo C.-J. (2017). The biophysicist’s guide to the bacterial flagellar motor. Adv. Phys. X.

[7] Smith A., Kaczmar A., Bamford R.A., Smith C., Frustaci S., Kovacs-Simon A., O’Neill P., Moore K., Paszkiewicz K., Titball R.W. (2018). The Culture Environment Influences Both Gene Regulation and Phenotypic Heterogeneity in Escherichia coli. Front. Microbiol..

[8] Hayes E.T., Wilks J.C., Sanfilippo P., Yohannes E., Tate D.P., Jones B.D., Radmacher M.D., BonDurant S.S., Slonczewski J.L. (2006). Oxygen limitation modulates pH regulation of catabolism and hydrogenases, multidrug transporters, and envelope composition in Escherichia coli K-12. BMC Microbiol..

[9] Cama J., Chimerel C., Pagliara S., Javer A., Keyser U.F. (2014). A label-free microfluidic assay to quantitatively study antibiotic diffusion through lipid membranes. Lab Chip.

[10] Cama J., Bajaj H., Pagliara S., Maier T., Braun Y., Winterhalter M., Keyser U.F. (2015). Quantification of Fluoroquinolone Uptake through the Outer Membrane Channel OmpF of Escherichia coli. J. Am. Chem. Soc..

[11] Díaz E., Ferrández A., Prieto M.A., García J.L. (2001). Biodegradation of Aromatic Compounds by *Escherichia coli*. Microbiol. Mol. Biol. Rev..

[12] Clarke P.H. (1982). The metabolic versatility of pseudomonads. Antonie Van Leeuwenhoek.

[13] Bishop K.J.M., Klajn R., Grzybowski B.A. (2006). The Core and Most Useful Molecules in Organic Chemistry. Angew. Chem. Int. Ed..

[14] Ratzke C., Gore J. (2018). Modifying and reacting to the environmental pH can drive bacterial interactions. PLoS Biol..

[15] Huertas M.J., Sáez L.P., Roldán M.D., Luque-Almagro V.M., Martínez-Luque M., Blasco R., Castillo F., Moreno-Vivián C., García-García I. (2010). Alkaline cyanide degradation by *Pseudomonas pseudoalcaligenes* CECT5344 in a batch reactor. Influence of pH. J. Hazard. Mater..

[16] Villano M., Beccari M., Dionisi D., Lampis S., Miccheli A., Vallini G., Majone M. (2010). Effect of pH on the production of bacterial polyhydroxyalkanoates by mixed cultures enriched under periodic feeding. Process Biochem..

[17] Vylkova S. (2017). Environmental pH modulation by pathogenic fungi as a strategy to conquer the host. PLoS Pathog..

[18] Farber J.M., Peterkin P.I. (1991). Listeria monocytogenes, a food-borne pathogen. Microbiol. Rev..

[19] Łapińska U., Glover G., Capilla-Lasheras P., Young A.J., Pagliara S. (2019). Bacterial ageing in the absence of external stressors. Philos. Trans. B.

[20] Nogales J., Mueller J., Gudmundsson S., Canalejo F.J., Duque E., Monk J., Feist A.M., Ramos J.L., Niu W., Palsson B.O. (2020). High-quality genome-scale metabolic modelling of *Pseudomonas putida* highlights its broad metabolic capabilities. Environ. Microbiol..

[21] Orth J.D., Conrad T.M., Na J., Lerman J.A., Nam H., Feist A.M., Palsson B.O. (2011). A comprehensive genome-scale reconstruction of *Escherichia coli* metabolism-2011. Mol. Syst. Biol..

[22] Sambrook J., Russell D.W. (2012). Molecular Cloning: A Laboratory Manual.

[23] Elbing K., Brent R. (2002). Media Preparation and Bacteriological Tools. Curr. Protoc. Mol. Biol..

[24] Nogales J., Agudo L. (2015). A Practical Protocol for Integration of Transcriptomics Data into Genome-Scale Metabolic Reconstructions.

[25] Nogales J., Gudmundsson S., Duque E., Ramos J.L., Palsson B.O. (2017). Expanding the Computable Reactome in *Pseudomonas putida* Reveals Metabolic Cycles Providing Robustness. bioRxiv.

[26] Nogales J., Gudmundsson S., Knight E.M., Palsson B.O., Thiele I. (2012). Detailing the optimality of photosynthesis in cyanobacteria through systems biology analysis. Proc. Natl. Acad. Sci. USA.

[27] Luque-Almagro V.M., Huertas M.J., Martinez-Luque M., Moreno-Vivian C., Roldan M.D., Garcia-Gil L.J., Castillo F., Blasco R. (2005). Bacterial degradation of cyanide and its metal complexes under alkaline conditions. Appl. Environ. Microbiol..

[28] Haynes W.M., Lide D.R., Bruno T.J. (2017). CRC Handbook of Chemistry and Physics.

[29] Escapa I.F., del Cerro C., García J.L., Prieto M.A. (2013). The role of GlpR repressor in *Pseudomonas putida* KT2440 growth and PHA production from glycerol. Environ. Microbiol..

[30] Gahlawat G., Soni S.K. (2017). Valorization of waste glycerol for the production of poly (3-hydroxybutyrate) and poly (3-hydroxybutyrate-co-3-hydroxyvalerate) copolymer by Cupriavidus necator and extraction in a sustainable manner. Bioresour. Technol..

[31] Randriamahefa S., Renard E., Guérin P., Langlois V. (2003). Fourier Transform Infrared Spectroscopy for Screening and Quantifying Production of PHAs by *Pseudomonas* Grown on Sodium Octanoate. Biomacromolecules.

[32] Igeño M.I., Macias D., Blasco R. (2019). A Case of Adaptive Laboratory Evolution (ALE): Biodegradation of Furfural by *Pseudomonas pseudoalcaligenes* CECT 5344. Genes.

[33] Manson M.D. (2020). pH Sensing in *Bacillus subtilis*: A New Path to a Common Goal. J. Bacteriol..

[34] Tohidifar P., Plutz M.J., Ordal G.W., Rao C.V. (2020). The Mechanism of Bidirectional pH Taxis in *Bacillus subtilis*. J. Bacteriol..

[35] Derek J., Chadwick G.C., Novartis Foundation (2007). Bacterial Responses to Ph.

[36] Sánchez-Clemente R., Igeño M.I., Población A.G., Guijo M.I., Merchán F., Blasco R. (2018). Study of pH Changes in Media during Bacterial Growth of Several Environmental Strains. Proceedings.

[37] Gale E.F., Epps H.M. (1942). The effect of the pH of the medium during growth on the enzymic activities of bacteria (*Escherichia coli* and *Micrococcus lysodeikticus*) and the biological significance of the changes produced. Biochem. J..

[38] Bartek I.L., Reichlen M.J., Honaker R.W., Leistikow R.L., Clambey E.T., Scobey M.S., Hinds A.B., Born S.E., Covey C.R., Schurr M.J. (2016). Antibiotic Bactericidal Activity Is Countered by Maintaining pH Homeostasis in *Mycobacterium smegmatis*. mSphere.

[39] Zarkan A., Caño-Muñiz S., Zhu J., Al Nahas K., Cama J., Keyser U.F., Summers D.K. (2019). Indole Pulse Signalling Regulates the Cytoplasmic pH of *E. coli* in a Memory-Like Manner. Sci. Rep..

[40] Ventola C.L. (2015). The antibiotic resistance crisis: Part 1: Causes and threats. Pharm. Ther..

[41] Molina L., Rosa R.L., Nogales J., Rojo F. (2019). *Pseudomonas putida* KT2440 metabolism undergoes sequential modifications during exponential growth in a complete medium as compounds are gradually consumed. Environ. Microbiol..

[42] Wolfe A.J. (2005). The Acetate Switch. Microbiol. Mol. Biol. Rev..

[43] Losen M., Frölich B., Pohl M., Büchs J. (2004). Effect of Oxygen Limitation and Medium Composition on *Escherichia coli* Fermentation in Shake-Flask Cultures. Biotechnol. Prog..

[44] Palsson B.O. (2015). Systems Biology. Constraint-Based Reconstruction and Analysis.

[45] Nikel P.I., de Lorenzo V. (2018). *Pseudomonas putida* as a functional chassis for industrial biocatalysis: From native biochemistry to trans-metabolism. Metab. Eng..

[46] Feist A.M., Palsson B.O. (2010). The biomass objective function. Curr. Opin. Microbiol..

[47] Naresh M., Das S., Mishra P., Mittal A. (2012). The chemical formula of a magnetotactic bacterium. Biotechnol. Bioeng..

[48] Popovic M. (2019). Thermodynamic properties of microorganisms: Determination and analysis of enthalpy, entropy, and Gibbs free energy of biomass, cells and colonies of 32 microorganism species. Heliyon.

